# Sleep of Children with High Potentialities: A Polysomnographic Study

**DOI:** 10.3390/jcm9103182

**Published:** 2020-09-30

**Authors:** Anne Guignard-Perret, Marine Thieux, Aurore Guyon, Stephanie Mazza, Min Zhang, Olivier Revol, Sabine Plancoulaine, Patricia Franco

**Affiliations:** 1 Pediatric Sleep Unit, Department of Pediatric Clinical Epileptology, Sleep Disorders and Functional Neurology, Hôpital-Femme-Mère-Enfant, Hospices Civils de Lyon, 59 boulevard Pinel, 69500 Lyon, France; anne.guignard-perret@chu-lyon.fr (A.G.-P.); marine.thieux@chu-lyon.fr (M.T.); aurore.guyon@chu-lyon.fr (A.G.); 2 INSERM U1028-CNRS UMR5292, Neuroscience Research Center, Centre Hospitalier Le Vinatier-Bâtiment 462 Neurocampus, 95 boulevard Pinel, University Lyon, 69675 Bron CEDEX, France; min.zhang@etu.univ-lyon1.fr; 3 HESPER laboratory, 8 Avenue Rockefeller, University of Lyon, 69003 Lyon, France; stephanie.mazza@univ-lyon1.fr; 4 Department of Developmental Psychopathology, Hôpital-Femme-Mère-Enfant, Hospices Civils de Lyon, 59 boulevard Pinel, 69500 Lyon, France; olivier.revol@chu-lyon.fr; 5 Université de Paris, CRESS, INSERM, INRAE, 16 Avenue Paul Vaillant Couturier 94807 Villejuif CEDEX, F-75004 Paris, France; sabine.plancoulaine@inserm.fr

**Keywords:** high potential, polysomnography, REM, sleep, anxiety, gifted children, intelligence quotient

## Abstract

The involvement of sleep in cognitive functioning is well known, but only a few studies have examined objective sleep parameters in children with high intellectual potential (HP). The main objective of this study was to compare sleep characteristics of 33 children with high intellectual potentialities (HP) (median 10 years old, 64% of boys) compared to 25 controls (median 11 years old, 64% of boys) and assess the difference between children with a homogeneous vs. a heterogeneous intelligence quotient (IQ) (i.e., a difference ≥15 points between verbal and non-verbal IQ). All children underwent a one-night polysomnography, an evaluation of intellectual quotient (IQ) and filled standardized questionnaires. Using non-parametric tests to compare groups’ characteristics, we found that children with HP had more heterogeneous IQ, more rapid eyes movement (REM) sleep and tended to have less stage 1 sleep than controls. They also had more insomnia and sleep complaints. The high amount of REM sleep in children with HP could be advantageous for learning and could partially explain their gift. This study highlights the necessity of investigating sleep disorders in children with HP during clinical routine and reinforces the hypothesis of the involvement of nocturnal sleep, and especially REM sleep, in daytime cognition and behavior.

## 1. Introduction

Nowadays, the most widely used measure of intelligence in children is based on the results of the Wechsler Intelligence Scale for Children (WISC) [1]. It determines a level of intelligence quotient (IQ) based on performances at several subdomains (verbal, perceptual reasoning, processing speed, working memory) compared to performances of same age peers [2]. Two types of IQ can be distinguished: homogeneous and heterogeneous (i.e., a difference ≥15 points between verbal and perceptual reasoning IQ) [3,4,5]. Intelligence in the general population follows a normal distribution, with slight excesses at the extremes represented by individuals with extremely poor or high cognitive abilities. An IQ ≥ 130 is commonly accepted to define high potentialities (HP) [6]. About 2.3% of children reach this criterion.

Various neurobiological markers have been associated with particular IQ differences. A positive correlation was shown between WISC subscales and spectral power of different Electroencephalogram (EEG) bands (especially in central and parietal regions), as well as between these subscales and fast EEG frequencies [7]. Features of sleep EEG may represent functional aspects of the underlying brain anatomy [8] including total brain volume, cortical thickness, white matter tract integrity, and brain activity during both task performance and rest [2]. A study showed a greater distribution of higher diffusivity regions in the right hemisphere in HP children with a homogeneous IQ and in the left hemisphere in those with a heterogeneous one. The left hemispheric specialization (HS) could underlie better verbal capacities but also self-centered behavior, while right HS could lead to greater adaptation capacities [5]. These changes in brain anatomy and brain functioning could result partly from genetic origin but also from environmental inputs. Indeed, a specific IQ depends on various factors such as motivation, health, sleep or socio-economic level of parents [2,9]. The involvement of sleep in humans cognitive processes [10], and especially in learning and memory [11], is supported by various hypotheses: the trace reactivation hypothesis suggests that sleep is involved in memory consolidation [12]; the synaptic homeostasis hypothesis suggests that sleep prevents saturation and preserves efficiency of neuronal networks [13]; and some evidences point out the role of sleep in maintaining functional integrity of the fronto-parietal networks [14]. With its slow-waves and spindles, non-rapid eyes movement (NREM) sleep represents the key-phenomena in these mechanisms. However, rapid eyes movement (REM) sleep per se might favor spreading the activation of a memory trace within cortical regions, leading to a reorganization of associative networks, helping to form new associations and reaching new solutions [15,16].

Despite their HP, children may display developmental asynchrony (intellectual development faster than affective or biological development) which can lead to repercussions on their daily life [3,6,17,18,19]. They commonly present mood and behavioral disorders, anxiety, somatization or social withdrawal and also are at risk for sleep disorders [20,21]. Indeed, HP children often complain about their sleep, especially difficulties falling asleep and night-awakenings, with a “short-sleeper” profile [21,22]. According to a French study, 17.4% of children with HP (*n* = 86, mean 9.3 years) have unspecified sleep disorders and 15.1% have enuresis [23]. However, these complaints are not always evaluated in clinical routine and almost never with objective measures.

Contrary to the large number of studies in children with intellectual or neurological disorders, only a few authors have examined objective sleep parameters in this population. There were only two studies in small samples of children and one in adolescents with HP. Moreover, these results were sparse and contradictory. The first study [24] looked at the sleep macrostructure of six children with HP compared to five controls (boys, aged 8–12 years). Compared to controls, children with HP displayed longer total sleep time, NREM stage 2 and NREM stage 3 (in min), and fewer eye movement density during REM sleep. There was a negative correlation between global IQ score and REM density. In another study, children with HP (*n* = 5) presented a higher duration of REM sleep than controls (*n* = 5, 10–13 years) [25]. More recently, Berdina et al., in a Russian journal, reported that adolescents with high intellectual abilities (evaluated by the Raven’s progressive matrices) (*n* = 44, 14–15 years) had higher percentage of REM sleep associated with a lower REM latency and a lower percentage of N3 [26].

The main objectives of this study were to analyze the subjective and objective sleep characteristics of a large group of children with HP compared to controls: (1) to assess the existence of a difference in brain function during sleep in these children; (2) to evaluate if there is a relationship with their sleep complaints; and (3) to compare these characteristics according to their homogeneous or heterogeneous cognitive profile.

## 2. Materials and Methods

### 2.1. Participants

Two groups of children were included in this retrospective case-control study: 33 children with HP and 25 control children. For both groups, inclusion criteria were: (a) age ≥ 5 and ≤ 15 years old, (b) ordinary school curriculum, (c) no major visual or auditory impairment, (d) no medical disease and (e) no psychiatric disorder. None had medical treatment at the time of the examination.

Children with HP (IQ ≥ 130) were recruited from both the ENSOM study (*n* = 4) (ENSOM N°2015-A00703-46, NCT02785328) and from the Reference Center for Gifted Children (*n* = 29) of the Department of Developmental Psychopathology, Hospices Civils de Lyon, France between 2013 and 2015. These children were referred to this center, essentially for school orientation.

Control children (IQ < 130) were recruited from the ENSOM study (*n* = 25) between 2015 and 2018, and were selected according to inclusion criteria.

### 2.2. Procedure

#### 2.2.1. General Data Collection

All children and parents had a systematic interview with a pediatric sleep specialist (P.F., A.G.-P.) in order to assess the presence of sleep disorders and to control the wake-sleep schedule before the recording night, as well as with a child psychiatrist (O.R.) to exclude psychopathological diagnoses. Information was collected on parental socio-economic level, on child demographics (age, sex) and school characteristics (presence of difficulties estimated by parents, grade repetition, and grade skipping, with a yes/no answer).

#### 2.2.2. Psychometric Assessment

Children’s psychometric assessments were performed by experienced neuropsychologists using the Wechsler intelligence scales according to the age: the Wechsler Preschool and Primary Scale of Intelligence (WPPSI) was used for 3 children (normalized from 4 to 7 years old) [27], while the WISC IV was used for other children (normalized from 7 to 16 years old) [1]. The IQ is the combination of the following 4 indexes: verbal comprehension index (VCI), perceptual reasoning index (PRI), working memory index (WMI) and processing speed index (PSI). Each score is standardized within each age group: the mean is 100 and the standard deviation is 15. The heterogeneity of the cognitive profile is defined by an absolute difference between VCI and PRI ≥ 15, known as the Significant Verbal Performance Discrepancy (SVPD). When the cognitive profile is heterogeneous, the IQ is not calculated and the general aptitude index (GAI), calculated from VCI and PRI, can be an alternative to IQ. GAI is less influenced by processing speed and working memory and is better suited to estimate the general cognitive functioning of children with HP [28]. Based on the WISC scores, children are classified as HP when they have a VCI, a PRI, an IQ and/or a GAI ≥ 130 (more than 2 SD from the mean of the normal distribution).

#### 2.2.3. Questionnaires

Three questionnaires were filled in, depending on the age, by parents or children under parental supervision: (a) Epworth Sleepiness Scale (ESS) for children: the most widely used scale which evaluates daytime sleepiness by an assessment of the risk of falling asleep in 8 daily life situations estimated on a 4-point Likert scale. The total score is the sum of the scores for the 8 items: a higher score represents greater sleepiness and the pathological threshold is higher than 10. The ESS has a good test-retest reliability (*r* > 89) and a good internal consistency (Cronbach α > 73) [29]; (b) Insomnia Severity Index (ISI): assessment of the insomnia severity in 7 items scored on a 5-point Likert scale ranging from “not at all” to “extremely.” The higher the total score, the more severe symptoms. The total score is considered pathological when it is higher than 10. The ISI has a good internal consistency (Cronbach α > 74) and a good reliability (*r* > 36) [30]; and (c) Child Depression Inventory (CDI): assessment of the depression symptomatology using 27 items scored on a 3-point Likert scale. The abnormal cut-off score is higher or equal to 16. The CDI has a good test-retest reliability (r > 38), a good internal consistency (Cronbach α > 80) and a good validity [31].

#### 2.2.4. Recordings

All children benefited from a single night ambulatory polysomnography (portative sleep recording system, DREAM, Medatec, Brussels, Belgium). The Polysomnography (PSG) was set up in the pediatric sleep department of the teaching hospital of Lyon (France) and children were sent home to sleep overnight. Parents were asked to supervise their child’s sleeping timetable (e.g., bedtime, lights-out, lights-on) as usual. Recordings were interrupted after awakening or at 8:00 a.m. maximum. The PSG included 8 encephalography electrodes referenced to the mastoids according to the 10–20 system, 2 electro-oculograms, a levator menti surface electromyography, inductance respiratory belts (thoracic and abdominal), and electrocardiography. The pediatric criteria of the American Sleep Disorders Association was used for the visual scoring of the PSG [32] by an experienced specialist (AGP). Total sleep time (TST), sleep and REM latency, sleep efficiency, duration and percentage of stage 1 (N1), stage 2 (N2), stage 3 (N3), REM sleep and wake after sleep onset (WASO), fragmentation and arousal indexes, number and mean duration of sleep cycles were collected.

### 2.3. Statistical Analysis

Statistical analyses were conducted by M.T. using the R software (version 3.6.3, Vienna, Austria) [33]. Continuous measures were expressed as median and range. Dichotomous and polytomous measures were expressed as *n* and percentage. Comparison between groups (children with HP vs. controls) were performed using unpaired Wilcoxon tests forcontinuous measures because of the non-normality of the distribution assessed by Shapiro-Wilk test. Fisher’s exact test was used for dichotomous measures. The same analysis was performed comparing children with HP with heterogeneous vs. homogeneous IQ. A false discovery rate (FDR) correction for multiple comparisons was used. Statistical significance value was set to a *p*-value below 0.05 for each test.

## 3. Results

Fifty-eight children were included in this retrospective case-control study: 33 children with HP and 25 controls without HP. There was no significant difference between groups concerning age, sex nor in socio-economic level of parents (*p* > 0.05). As suggested by our groups’ repartition, children with HP had significantly higher WISC scores than controls, except for PSI and with a trend for WMI (Table 1). The frequency of heterogeneous IQ was higher in children with HP than in controls (60% vs. 24%, *p* = 0.04).

Twenty children had at least one sleep complaint: 15 had insomnia (associated with enuresis in one child and with phase shifts in another), two had night terrors (associated with enuresis in one case), two had phase shifts and one had complaints for agitated sleep. Sleep complaints were more common in children with HP than in controls (52% vs. 12%, *p* = 0.02).

Nine children with HP experienced grade skipping, while it never occurred in control children (33% vs. 0%, *p* = 0.02). Children with HP tended to report school difficulties more frequently, but not significantly, than controls (44% vs. 18%, *p* = 0.13). There was no difference between the two groups concerning grade repetition (*p* = 0.31).

Children with HP had more common pathological scores at the ISI than controls (55% vs. 14%, *p* = 0.02). The ISI median score was also higher in children with HP compared to controls. There was no difference between groups concerning the Epworth (7% vs. 15%, *p* = 0.72) and the CDI (43% vs. 23%, *p* = 0.31) scores (Table 1).

**Figure 1:**
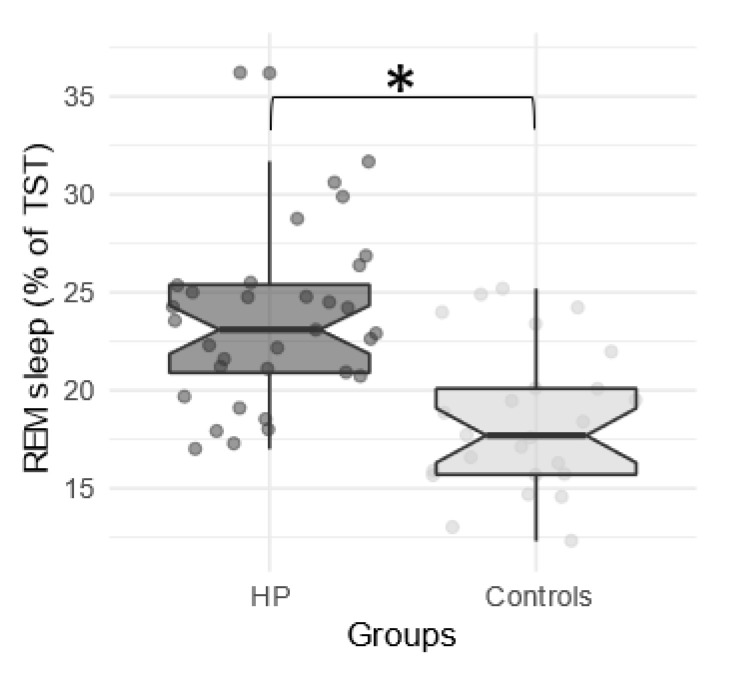
Each point represents the median Rapid Eye Movement (REM) sleep percentage for each subject according to the group (High Potential (HP) vs. controls). The central line of the boxplot corresponds to the median; the upper and lower parts correspond to the first and third quartiles. Difference between groups is represented by a star: * *p* < 0.05.

Concerning sleep characteristics, both duration and proportion of REM sleep (expressed as percentage of TST) were significantly higher in children with HP compared to controls (Figure 1), while stage 1 (N1) sleep duration and percentage tended to be lower in children with HP. Analyses of main parameters of sleep continuation (TSP, TST, efficiency, latency and REM latency, WASO, fragmentation index and micro-arousal index) did not show any significant difference between groups. Both duration and proportion of N2 and N3 as well as the number and duration of sleep cycles did not reveal any significant difference (Table 2).

Comparing children with HP with heterogeneous or homogeneous IQ, we found that even if sleep complaints were more common in children with a heterogeneous IQ compared to those with a homogeneous one, statistical significance was not reached (65% vs. 31%, *p* = 0.52). The most common complaint was insomnia in 12 children (associated with enuresis in one child, with phase shifts in another one). There were also two complaints for night terrors (with an association with enuresis in one case), two complaints for phase shifts and one complaint for agitated sleep. However, there were no other differences between these groups concerning demographic, questionnaire nor sleep characteristics evaluated by the PSG.

## 4. Discussion

First of all, children with HP frequently expressed sleep complaints. Insomnia is the most commonly reported sleep disorder and children with HP presented pathological ISI scores more frequently than controls. It was previously shown that, compared to a control population, children with HP have more sleep disorders [23], especially difficulties in falling asleep [21,22]. These complaints might be related to anxiety which is overrepresented in children with HP [34,35]. Differences in hemispheric specialization (HS) were suggested between children with HP according to their cognitive profile in favor of a right HS in those with a homogeneous IQ. The right HS could underlie greater capacities of adaptation [5], leading to less anxiety and sleep disorders. In our study, even if the children with heterogeneous IQ reported more sleep complaints, no significant differences were found between these children and those with a homogenous profile after accounting for multiple testing. This could be due to the small number of children.

In our study, no significant difference was found concerning sleep characteristics measured by PSG between HP children according to their cognitive profile. Moreover, no significant difference was found between HP children and controls for most sleep parameters including cycle number and duration, arousals or REM and sleep latency, although HP children tended to have lower percentage of N1, which could reflect a better sleep quality than controls. PSG parameters between HP and controls did not differ drastically except for REM sleep. Except in Busby & Pivik’s study based on a small sample of children [24], our results are in line with some previous studies [25,26,36]. A relationship between REM sleep and cognition was previously reported in children with intellectual deficits and in gifted children [25,26,36]. Indeed, a low percentage of REM sleep was found in 54 children (9.8 years) with intellectual deficits compared to 17 controls (9.0 years), with a negative correlation between REM sleep and IQ parameters. Inversely, a higher percentage of REM sleep was shown in children with HP compared to controls [25]. The recent Russian study conducted by Berdina et al. confirmed that adolescents with high intellectual abilities had a higher percentage of REM sleep [26].

The high amount of REM sleep in HP children could be understood in phylogenetic and ontogenetic ways, with studies showing that more immature species on neurodevelopment at birth have a higher percentage of REM sleep than the more mature ones [25,37,38]. Human newborns have more than 50% of REM sleep and this percentage decreases progressively during the first years of life, reaching 20% in adults. This percentage of REM sleep could be a marker of cerebral plasticity, helping the subject to collect and store information [38,39,40,41,42], which is particularly useful in memory consolidation and learning [39,40,42,43].

Studies in functional MRI suggest that children with HP have brain networks with less segregation, less modularization and more global integration than controls. In children with HP, the connectivity is especially high in the associative cortex. The inter- and intra-hemispheric white matter integrity are also enhanced in these children [5,44]. Since problem-solving and creativity involve the associative cortex during REM sleep, HP children could be favored compared to controls. This higher amount of REM sleep could partially explain their gift.

In a neuropsychological view, we could hypothesize that children with higher IQ have more REM sleep during the night because of their brain functioning and that could be advantageous for learning. In clinical routines, we have to consider that the cognitive profile may influence the perception of sleep reflected by higher ISI scores in HP without altering sleep quality. In this perspective, when the HP is diagnosed, it is necessary to investigate the existence of sleep disorders and consider a possible cognitive-behavioral or psychological therapy to overcome anxiety and sleep disorders, and thus offer appropriate support to these children.

To the best of our knowledge, this is the first comparative study of sleep macrostructure in HP children based on a large sample. However, our study has some limitations. First of all, only one night of PSG data was collected. Thus, these acute measurements of sleep may not reflect general and usual characteristics. However, children had the opportunity to sleep at home in a less stressful environment than a sleep lab. These conditions were reflected by the good sleep parameters (i.e., sleep duration, good sleep efficiency and low sleep fragmentation indexes). Herein, we did not record the respiratory parameters during PSG. However, no children in our study presented Obstructive Sleep Apnea (OSA) clinical symptoms or paradoxal breathing on thoraco-abdominal belts. On the other hand, the respiratory parameters like nasal canulae or thermistors were more susceptible to altering sleep structure or to be removed in an ambulatory recording. Another limitation is due to the cross-sectional design of the study, which cannot permit us to draw a causality relationship between IQ and REM sleep. Moreover, it was not possible on this limited sample to conduct multivariate analyses which could lead to a better understanding of the relationship between the cognitive profile, mood and sleep parameters. A quantitative and spectral analysis of sleep structure could also be relevant to deepen our understanding of the relation between sleep and cognitive performances.

## 5. Conclusions

This study reinforces the hypothesis of the involvement of nocturnal sleep, and especially REM sleep, in daytime cognition and behavior and highlights the necessity of investigating sleep disorders in children with HP in clinical routine.

## Figures and Tables

**Table 1:** Demographic, questionnaires and neuropsychological characteristics for children with HP and controls.

	Controls		HP		*P*_Wilcoxon_
	***n* = 25**		***n* = 33**		
		missing		missing	
**Demographic characteristics**					
Sex (male), (n, percentage)	16 (64)	0	21 (64)	0	1.00
Age at the PSG, years (median) [min-max]	10 [5.5–15]	0	11 [6.5–15]	0	0.39
**Neuropsychological characteristics**					
VCI (median) [min–max]	114 [82–128]	0	143 [122–155]	0	<0.001
PRI (median) [min–max]	104 [77–124]	0	128 [96–148]	0	<0.001
WMI (median) [min–max]	100 [60–133]	0	111 [79–133]	3	0.07
PSI (median) [min–max]	106 [64–143]	0	106 [86–143]	2	0.58
GAI (median) [min–max]	110 [80–125]	2	139 [121–156]	2	<0.001
IQ (median) [min–max]	111 [89–127]	6	139 [115–147]	13	<0.001
VCI-PRI ≥ 15 (n, percentage)	7 (24)	0	20 (60)	0	0.04
**Questionnaires**					
ISI (median) [min–max]	4 [1–17]	4	11 [0–19]	6	0.03
CDI (median) [min–max]	10 [1–25]	4	11 [0–36]	5	0.63
Epworth (median) [min–max]	2 [0–16]	2	3 [0–21]	4	0.76

HP: high potential; PSG: polysomnography; VCI: verbal comprehension index; PRI: perceptual reasoning index; WMI: working memory index; PSI: processing speed index; GAI: general ability index; IQ: intellectual quotient; ISI: insomnia severity index; CDI: children depression inventory.

**Table 2:** Characteristics of the Polysomnography (PSG) for children with HP and controls.

	Controls		HP		*p*-Value
	***n* = 25**		***n* = 33**		
		missing		missing	
	median [min–max]		median [min–max]		
Total sleep period (TSP), min	564 [395–671]	0	541 [479–633]	1	0.56
Total sleep time (TST), min	528 [348–655]	0	522 [349–623]	0	0.57
Sleep efficiency, %	95.7 [82.7–98]	0	96 [68–98.7]	1	0.57
Sleep latency, min	16 [6–61]	0	22 [6–103]	2	0.57
REM latency, min	148 [50–243]	0	143 [56–209]	0	0.51
N1, min	58 [27–168]	0	54 [23–93]	1	0.09
N1, %	12.1 [5.1–30.8]	0	10.1 [4.4–18.1]	1	0.05
N2, min	256 [140–299]	0	234 [137–333]	1	0.26
N2, %	45.5 [30.1–57.4]	0	43.8 [19.4–56.9]	1	0.36
N3, min	115 [73–149]	0	111 [65–151]	0	0.69
N3, %	22 [13.3–35.3]	0	21.3 [12.1–29]	0	0.81
REM sleep, min	100 [62–143]	0	124 [64–225]	0	0.009
REM sleep, %	17.7 [12.3–25.2]	0	23.1 [17–36.2]	0	<0.001
WASO	22 [9–88]	0	21.5 [7–118]	1	0.58
Fragmentation index/h	10.3 [2.1–17.7]	0	11.9 [6.3–18.4]	1	0.22
Arousal index/h	8.7 [4.8–14.8]	0	9.3 [4.6–15.7]	1	0.82
Number of cycles	5 [3–6]	0	5 [4–6]	1	0.56
Mean cycle duration, min	105 [81–126]	1	98 [77–131]	5	0.27

HP: high potential; N1: stage 1; N2: stage 2; N3: stage 3; REM: rapid eyes movements; WASO: wake after sleep onset; h: hour; min: minutes.

## Abbreviations

Child Depression Inventory (CDI); Electrocardiogram (ECG); Electroencephalogram (EEG); Electromyogram (EMG); Electrooculogram (EOG); General Ability Index (GAI); Hospital Anxiety and Depression Scale (HAD); High Potential (HP) Insomnia Severity Index (ISI); Intellectual Quotient (IQ); Multiple Imputation with Chained Equations (MICE); Obstructive Sleep Apnea Syndrome (OSA); Perceptual Reasoning Index (PRI); Polysomnography (PSG); Processing Speed Index (PSI); Rapid Eye Movement sleep (REM); Significant Verbal Performance Discrepancy (SVPD); Stage 1 (S1); Stage 2 (S2); Stage 3 (S3); Total Sleep Period (TSP); Total Sleep Time (TST); Verbal Comprehension Index (VCI); Verbal Performance Discrepancy (VPD); Wake After Sleep Onset (WASO; Working Memory Index (WMI).

## Clinical Trials

ENSOM: https://clinicaltrials.gov/ct2/show/NCT02785328?cond=sleep&cntry=FR&city=lyon&rank=4.
